# Avaliação transversal do eixo mecânico e demais parâmetros angulares dos membros inferiores em atletas profissionais de futebol do sexo masculino

**DOI:** 10.1055/s-0046-1818619

**Published:** 2026-04-22

**Authors:** Jean Klay Santos Machado, Eurineto Gomes do Nascimento, Erik Silva de Menezes

**Affiliations:** 1 Serviço de Ortopedia e Traumatologia, Hospital Porto Dias, Belém, PA, Brasil

**Keywords:** fenômenos biomecânicos, futebol, medicina esportiva, biomechanical phenomena, soccer, sports medicine

## Abstract

**Objetivo:**

Avaliar o eixo mecânico e os parâmetros angulares dos membros inferiores em atletas profissionais de futebol, relacionando-os à idade, dominância lateral e posição em campo.

**Métodos:**

Conduzimos um estudo transversal com 102 atletas masculinos de um clube. Radiografias panorâmicas em ortostase foram analisadas pelo programa PeekMed (Peek Health, S.A.), que mensurou o parâmetro de eficiência condilar (PEC), o ângulo mecânico medial proximal tibial (AmMPT), o ângulo mecânico lateral distal femoral (AmLDF) e o ângulo mecânico tibiofemoral (AmTF). Aplicaram-se estatística descritiva, analise de variância ( *analysis of variance* , ANOVA, em inglês), análise multivariada da variância ( *multivariate analysis of variance* , MANOVA, em inglês), correlações de Pearson/Spearman e regressão múltipla.

**Resultados:**

A idade média foi de 26,9 anos. O valgo foi o padrão mais prevalente (68,6%), com bilateralidade em 47,0%. Houve correlação negativa entre idade e PEC bilateral (ρ = -0,42 à direita; e -0,39 à esquerda) e maior assimetria condilar (ΔPEC) em zagueiros e canhotos. A MANOVA indicou associação significativa entre posição e parâmetros articulares ( *p* = 0,0016), sem efeito global da dominância ( *p* = 0,243). Laterais e meias apresentaram maior prevalência de valgo (até 80%), ao passo que zagueiros e goleiros mostraram mais casos de eixo neutro. Diferenças bilaterais significativas foram observadas com relação ao PEC, ao AmMPT e ao AmLDF.

**Conclusão:**

O alinhamento dos membros inferiores em atletas de futebol mostrou-se influenciado pela idade e posição em campo, com assimetrias mais marcantes em zagueiros e canhotos. O monitoramento individualizado do eixo mecânico pode auxiliar na prevenção de lesões e otimização do desempenho.

## Abstract (en)

**Objective:**

To evaluate the mechanical axis and angular parameters of the lower limbs in professional soccer players, relating them to age, limb dominance, and field position.

**Methods:**

The present cross-sectional study included 102 male athletes from a Brazilian professional soccer team. Full-length radiographs in the orthostatic position were analyzed using the PeekMed (Peek Health, S.A.) software to measure the condylar efficiency (CE), the medial mechanical proximal tibial angle (mMPTA), the mechanical lateral distal femoral angle (mLDFA), and the mechanical tibiofemoral angle (mTFA). The results were analyzed using descriptive statistics, analysis of variance (ANOVA), multivariate analysis of variance (MANOVA), Pearson's/Spearman's correlations, and multiple regression.

**Results:**

The mean age of the participants was of 26.9 years. The valgus pattern was the most prevalent (68.6%), with bilateral valgus in 47.0% of the sample. A negative correlation was found between age and bilateral CE (ρ = -0.42 on the right side; and -0.39 on the left side; p < 0.05), and defenders and left-footed players presented greater condylar asymmetry (ΔCE). The MANOVA indicated a significant association between field position and angular parameters ( *p* = 0.0016), with no global effect of dominance ( *p* = 0.243). Midfielders and fullbacks showed a higher prevalence of valgus (of up to 80%), while defenders and goalkeepers commonly presented with neutral alignment. There were significant bilateral differences in CE, mMPTA, and mTFA.

**Conclusion:**

Age and field position influenced lower-limb alignment in professional soccer players, with more pronounced asymmetries in defenders and left-footed athletes. Individualized monitoring of the mechanical axis may help prevent injuries and optimize performance.

## Introdução

O alinhamento coronal dos membros inferiores é essencial na ortopedia esportiva, especialmente em atletas expostos a altas cargas biomecânicas. Alterações no eixo mecânico podem gerar sobrecarga articular, predispor a lesões e comprometer o desempenho. Tradicionalmente, o joelho é classificado em neutro, varo ou valgo, porém essa divisão não reflete a complexidade anatômica individual. 1

O joelho neutro corresponde a um ângulo quadril-joelho-tornozelo ( *hip-knee-ankle* , HKA, em inglês) de 180°, com linhas articulares femoral e tibial oblíquas em 3° (varo na tíbia e valgo no fêmur), que resulta em um ângulo femoral mecânico ( *femoral mechanical angle* , FMA, em inglês) de 93° e o ângulo tibial mecânico ( *tibial mechanical angle* , TMA, em inglês) de 87°, ambos medidos medialmente. 1

O alinhamento é determinado por fatores anatômicos e mecânicos, principalmente pelas linhas articulares femorais e tibiais. 2 Entre os parâmetros utilizados, destacam-se o ângulo femoral distal anatômico lateral ( *lateral anatomical distal femoral angle* , LaDFA, em inglês), o ângulo tibial proximal medial ( *medial proximal tibial angle* , MPTA, em inglês) e o ângulo do quadríceps (ângulo Q), considerados indicadores relevantes na avaliação da retidão em atletas de futebol. 3 Variações nesses ângulos podem predispor a lesões e comprometer o desempenho esportivo. 3

O desvio do eixo mecânico ( *mechanical axis deviation* , MAD, em inglês), avaliado pela radiografia panorâmica, pode gerar sobrecarga assimétrica no joelho. O varo (eixo medializado) associa-se a lesões no compartimento medial, e o valgo (eixo lateralizado), a lesões laterais. 4 5 A radiografia panorâmica é o padrão-ouro, e fornece medidas objetivas e reprodutíveis. 6 A precisão depende da identificação correta dos pontos anatômicos de referência. 7 O eixo mecânico é um conceito central da biomecânica do joelho, que influencia a distribuição de cargas articulares e o risco de lesões. Seu reconhecimento e correção são fundamentais na prevenção e no tratamento de lesões esportivas. 4 6

Com base nas considerações apresentadas, este estudo teve como objetivo avaliar o eixo mecânico e outros parâmetros angulares dos membros inferiores em atletas profissionais de futebol masculino, relacionando-os à idade, à dominância lateral e à posição funcional em campo.

## Métodos

### Aspectos Éticos

O estudo foi aprovado pelo Comitê de Ética em Pesquisa institucional em conformidade com a Declaração de Helsinque, o Código de Nuremberg e a Resolução n° 466/12 do Conselho Nacional de Saúde (CNS) sob o número CAAE: 84206924.5.0000.0235. Todos os participantes assinaram o termo de consentimento livre e esclarecido. Os riscos potenciais, como o de vazamento de dados e o da exposição em ambiente hospitalar, foram minimizados por meio da anonimização das informações, do armazenamento seguro dos registros e do cumprimento rigoroso das normas de biossegurança recomendadas pela Organização Mundial da Saúde. Como benefício, o estudo ampliou o conhecimento sobre o alinhamento mecânico em atletas profissionais, o que subsidia futuras pesquisas e intervenções preventivas.

### Participantes, Local da Pesquisa e Justificativa do Tamanho Amostral

O estudo foi realizado em hospital de alta complexidade da Região Norte, entre janeiro de 2023 e dezembro de 2024, com 102 atletas masculinos profissionais de futebol de campo, incluindo todos os jogadores do clube no período. A amostra representou a população-alvo de atletas de alto rendimento e mostrou-se adequada para análises multivariadas (análise de variância [ *analysis of variance* , ANOVA, em inglês], análise multivariada de variância [ *multivariate analysis of variance* , MANOVA, em inglês], regressão múltipla e correlações), em consonância com estudos prévios com amostras de 30 a 100 indivíduos.

O tamanho amostral ( *n* = 102) foi avaliado quanto ao poder estatístico por meio do programa R (R Foundation for Statistical Computing), com o pacote pwr, em modelo de regressão múltipla com 3 preditores (idade, posição e dominância lateral). Adotou-se nível de significância de 5% (α = 0,05) e tamanho de efeito moderado (f ^2^ = 0,15), conforme os critérios de Cohen. 8 Os resultados indicaram que a amostra apresentou poder estatístico adequado para detectar associações de magnitude moderada entre as variáveis analisadas, o que atende aos critérios de robustez metodológica recomendados na literatura.

### Coleta e Análise de Dados

A coleta ocorreu entre janeiro de 2023 e dezembro de 2024, por questionário eletrônico (Google Forms, Alphabet Inc.) e exames radiográficos padronizados, e 102 atletas enviaram respostas completas. Foram excluídos participantes com formulários incompletos ou falhas nas etapas radiográficas, sem dados faltantes na amostra final. As informações foram organizadas em planilhas no Microsoft Excel 2016 (Microsoft Corp.).

Para a análise do eixo mecânico dos membros inferiores, utilizaram-se radiografias panorâmicas em ortostase com apoio bipodálico, processadas por ortopedista membro da Sociedade Brasileira de Ortopedia e Traumatologia (SBOT) e da Sociedade Brasileira de Cirurgia de Joelho (SBCJ), com auxílio do programa PeekMed (Peek Health, S.A.). O processo envolveu calibração com esfera metálica de 25 mm e identificação de pontos anatômicos de referência (cabeça femoral, platô tibial e tálus). A partir desses pontos, traçou-se o eixo mecânico, o que permitiu a análise objetiva dos desalinhamentos em varo ou valgo. Considerou-se normalidade do eixo mecânico variação entre 0° e 2°, tanto para valgo quanto varo.

**Fig. 1:**
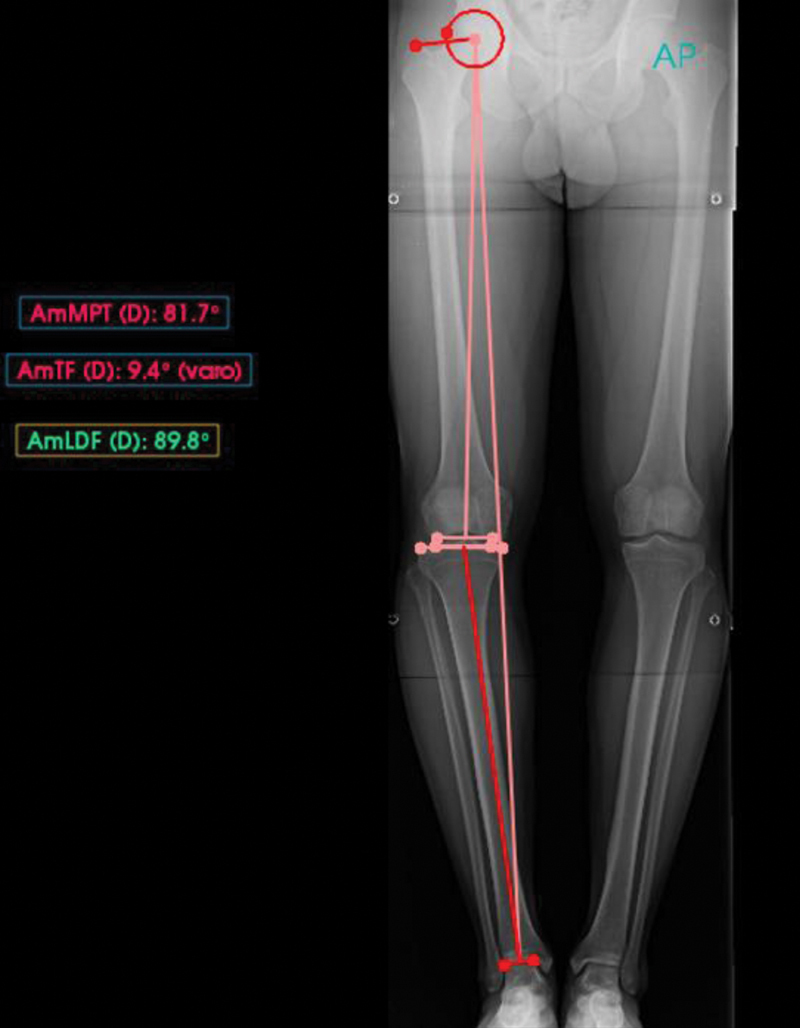
Radiografia panorâmica de membros inferiores com mensurações angulares.

O PeekMed forneceu automaticamente as medidas de cada membro inferior, conforme descrição das Figs. 1 2 :

**Fig. 2:**
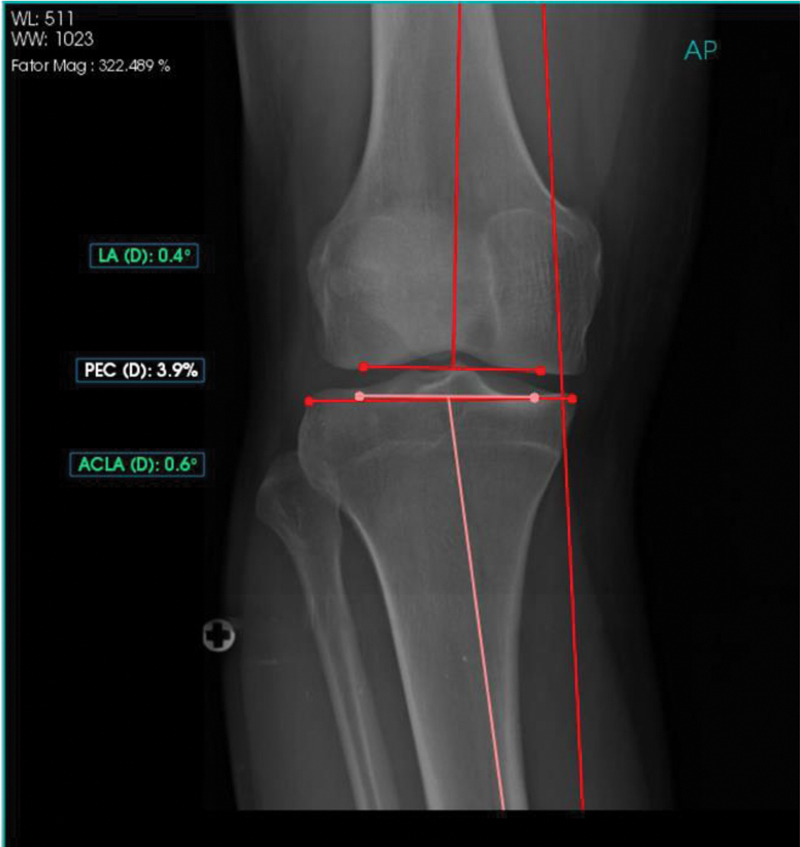
Radiografia de joelho em incidência anteroposterior (AP) com mensurações angulares.

- MAD (em milímetros);
- Ângulo HKA: ângulo entre os eixos mecânicos do fêmur e da tíbia;
*- Lateral distal femoral angle* (LDFA): ângulo lateral do fêmur distal; e
- MPTA.

Todas as medidas obtidas foram registradas e exportadas em formato digital e analisadas no PeekMed, e foram asseguradas precisão e reprodutibilidade das mensurações. As variáveis foram expressas por valores de média, mediana e desvio padrão. A normalidade da amostra foi avaliada pelo teste de Shapiro-Wilk, e as correlações, pelos coeficientes de Pearson ou Spearman. Para a comparação entre os grupos, aplicaram-se MANOVA, ANOVA ou o teste de Kruskal-Wallis, de acordo com a distribuição de dados. Modelos de regressão múltipla investigaram a influência de idade, posição em campo e dominância sobre os ângulos. Para comparação entre lados direito e esquerdo, utilizaram-se testes pareados ( *t* de Student ou de Wilcoxon). O nível de significância estatística adotado foi de 5% ( *p* < 0,05).

## Resultados

Foram avaliados 102 jogadores profissionais de futebol, com idade média de 26,9 ± 5 anos, faixa típica de atletas em atividade plena. Destes, 81% chutavam com a perna direita (destros) e 19%, com a esquerda (canhotos). Quanto à posição em campo, 31% eram atacantes, 21%, meias, 18%, zagueiros, 15%, laterais, 12%, volantes, e 4%, goleiros.

**Fig. 3:**
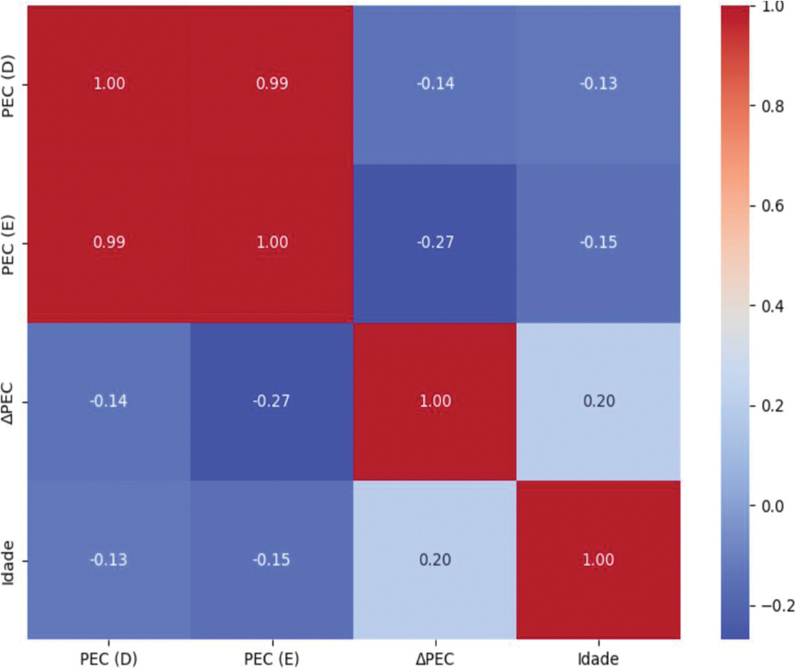
Correlograma de correlações de Spearman entre idade e variáveis condilares (o parâmetro de eficiência condilar direito [PEC-D], PEC esquerdo [PEC-E] e assimetria condilar [ΔPEC]). Os coeficientes estão indicados nas células, com coloração proporcional à intensidade da correlação.

A análise de correlação de Spearman mostrou associação negativa entre idade e o parâmetro de eficiência condilar (PEC), tanto no joelho direito (ρ = -0,42; *p* = 0,002) quanto no esquerdo (ρ = -0,39; *p* = 0,005), o que indica que atletas mais velhos apresentaram valores menores desse parâmetro, conforme a Fig. 3 . O PEC reflete o alinhamento e a eficiência do contato fêmur-tíbia, sendo que valores reduzidos sugerem maior risco de desequilíbrio articular. Também foi observado aumento progressivo do ângulo tibiofemoral esquerdo ( *left tibiofemoral angle* , LTFA, em inglês) com a idade (ρ = +0,28; *p* = 0,01), o que sugere possível adaptação ou sobrecarga no alinhamento do joelho ao longo do tempo ( Tabela 1 ).

**Tabela 1:** Correlação entre idade e ângulos

Variável	Coeficiente (r/ρ)	Valor de *p*	Tipo
PEC (D)	-0,42	0,002	Spearman
PEC (E)	-0,39	0,005	Spearman
mTFA (E)	+0,28	0,01	Spearman

Abreviaturas: D, direito; E, esquerdo; mTFA, *mechanical tibiofemoral angle* , (ângulo tibiofemoral mecânico); PEC, parâmetro de eficiência condilar.

A regressão linear múltipla para o PEC do lado direito identificou três preditores significativos: idade (β = -0,38; *p* = 0,004), dominância canhota (β = +1,25; *p* = 0,049) e a posição de zagueiro (β = +2,40; *p* = 0,021). O modelo apresentou coeficiente de determinação ajustado de R ^2^ = 0,27, o que indica que esses fatores explicam aproximadamente 27% das variações do PEC ( Tabela 2 ).

**Tabela 2:** Modelo para o parâmetro de eficiência condilar direito como variável dependente

Variável	Coeficiente (β)	Valor de *p*
Idade	-0,38	0,004
Dominância (canhoto)	+1,25	0,049
Posição: zagueiro	+2,40	0,021
R ^2^ ajustado	0,27	−

**Fig. 4:**
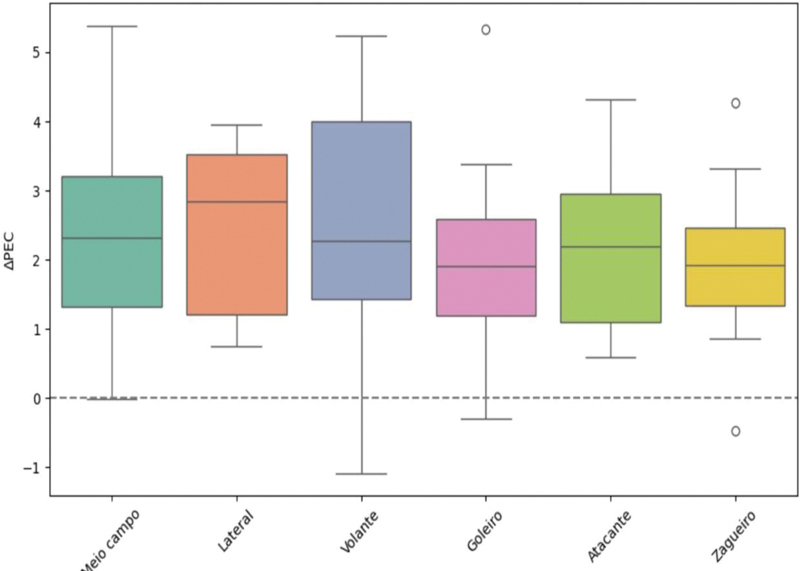
Distribuição do ΔPEC segundo a posição dos atletas. Variações maiores são observadas entre laterais, meias e volantes.

Na MANOVA, a posição em campo apresentou associação significativa com as variáveis biomecânicas (λ de Wilks = 0,69; F = 2,42; *p* = 0,0016). A dominância lateral não demonstrou significância estatística (λ de Wilks = 0,92; F = 1,21; *p* = 0,243) ( Tabela 3 e Fig. 4 ).

**Tabela 3:** Análise multivariada de variância

Fator	λ de Wilks	F	Valor de *p*
Posição	0,69	2,42	0,0016
Dominância	0,92	1,21	0,243

O PEC apresentou valor 2,1% maior no joelho direito em relação ao esquerdo ( *p* = 0,011). O MPTA mecânico ( *mechanical* MPTA, mMPTA, em inglês; o ângulo de inclinação da parte superior da tíbia) mostrou diferença de +1,0° no lado direito ( *p* = 0,044). Já o LDFA mecânico ( *mechanical* LDFA, mLDFA, em inglês) foi 0,7° menor no lado direito, o que corresponde a valor mais alto no lado esquerdo ( *p* = 0,033) ( Tabela 4 ). As diferenças bilaterais (Δ) apresentaram correlação negativa significativa entre idade e ΔPEC (ρ = -0,31; *p* = 0,006) e entre idade e ΔmMPTA (ρ = -0,24; *p* = 0,027) ( Tabela 5 ).

**Tabela 4:** Diferença bilateral (direita e esquerda) dos ângulos

Variável	Teste	Diferença média	Valor de *p*
PEC (%)	Wilcoxon	+2,1%	0,011
mMPTA (°)	*t* pareado	+1,0°	0,044
mLDFA (°)	*t* pareado	-0,7°	0,033

Abreviaturas: D, direito; E, esquerdo; mMPTA, *mechanical medial proximal tibial angle* (ângulo ângulo tibial proximal tibial medial mecânico); mLDFA, *mechanical lateral distal femoral angle* (ângulo femoral distal lateral mecânico); PEC, parâmetro de eficiência condilar.

**Tabela 5:** Correlação de ΔPEC e ΔAmMPT com idade

Diferença	Coeficiente de Spearman	Valor de *p*
ΔPEC	-0,31	0,006
ΔmMPTA	-0,24	0,027

Abreviaturas: mMPTA, *mechanical medial proximal tibial angle* (ângulo ângulo tibial proximal tibial medial mecânico); PEC, parâmetro de eficiência condilar.

**Fig. 5:**
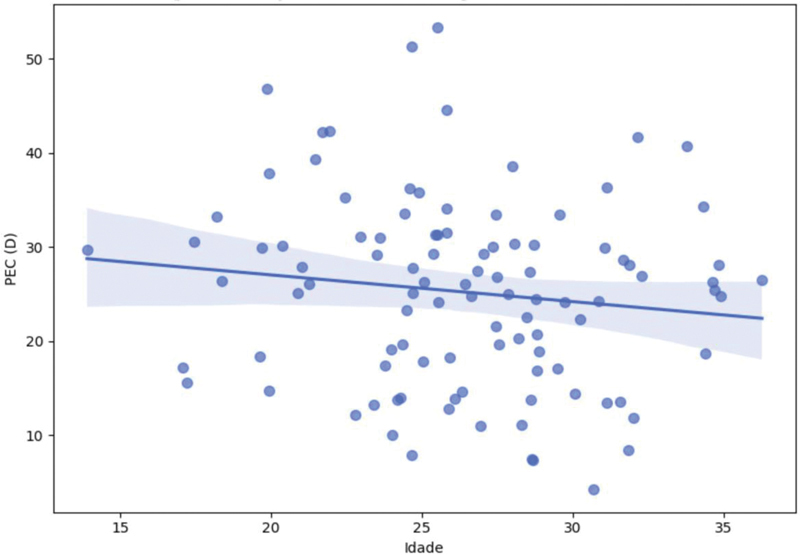
Relação entre idade e o PEC-D. A linha de tendência indica correlação negativa com IC95%.

No modelo de regressão múltipla com ΔPEC como variável dependente, os preditores estatisticamente significativos foram: idade (β = -0,33; *p* = 0,008), o que indica menor assimetria em atletas mais velhos (β = +1,4; *p* = 0,041), associada a maior assimetria; e posição de zagueiro (β = +1,9; *p* = 0,019), também relacionada a maiores diferenças. O modelo apresentou R ^2^ ajustado de 0,22 ( Tabela 6 e Fig. 5 ).

**Tabela 6:** Modelo preditivo para assimetria condilar

Variável	Coeficiente (β)	Valor de *p*
Idade	-0,33	0,008
Canhoto	+1,4	0,041
Zagueiro	+1,9	0,019
R ^2^ ajustado	0,22	−

Na comparação entre os lados direito (D) e esquerdo (E), o ângulo de valgo/varo apresentou diferença significativa, com média de 1,2° no D e 1,5° no E ( *p* = 0,045), o que sugere discreto aumento no E. A flexão do joelho apresentou médias de 87,5° (D) e 86,8° (E), sem diferença significativa.

Na correlação com a idade, não houve associação significativa com o ângulo de valgo/varo (D: r = 0,15; *p* = 0,12). Porém, identificou-se correlação negativa entre idade e flexão do joelho D (r = -0,22; *p* = 0,03), o que indica menor amplitude de flexão em atletas mais velhos.

A ANOVA mostrou diferença significativa no ângulo de valgo/varo entre posições em campo (D: F [4,95] = 2,89; *p* = 0,027), com laterais apresentando, em média, maiores valores angulares. Para flexão do joelho, não houve diferença significativa entre posições (D: F [4,95] = 1,76; *p* = 0,14).

O teste *t* independente mostrou ausência de diferença significativa entre destros e canhotos para o ângulo de valgo/varo ( *p* = 0,38) e para a flexão do joelho D ( *p* = 0,22), o que indica que a dominância lateral não influenciou os parâmetros articulares analisados.

## Discussão

Este estudo analisou a relação entre ângulos ortopédicos do membro inferior e fatores clínicos e funcionais em atletas profissionais, com foco nos ângulos mecânicos tibiofemorais e de carga condilar, considerando idade, posição funcional e dominância lateral.

### Idade e Alterações Articulares

Evidenciou-se correlação negativa entre idade e o PEC, o que indica menor adaptação articular em atletas mais velhos. O achado confirma a tendência descrita por Steffen et al., 9 de aumento progressivo do varo com o avanço etário e sobrecarga cumulativa. Davidoviča et al. 10 observaram alterações biomecânicas precoces no quadril e no joelho, o que sugere que o remodelamento condilar em atletas profissionais decorre da progressão dessas adaptações. O envelhecimento esportivo associa-se à redução da eficiência condilar e à modificação do eixo mecânico, o que reforça a necessidade de monitoramento contínuo dessas variáveis.

### Efeitos da Posição Funcional

A posição em campo influenciou significativamente os ângulos avaliados (λ de Wilks = 0,69; *p* = 0,0016), com zagueiros e laterais apresentando maior assimetria condilar. Achado semelhante foi descrito por Steffen et al., 9 que observaram maior varo em defensores, e por Ribeiro et al., 11 que destacaram o caráter multifatorial das adaptações posicionais. Murillo-Ortiz et al. 12 também relataram assimetrias decorrentes de gestos unilaterais repetitivos. Assim, confirma-se que a função tática e a carga específica da posição modulam o eixo mecânico e promovem adaptações osteoarticulares ao longo do tempo.

### Dominância Lateral

Não houve diferença global significativa entre destros e canhotos na MANOVA. Entretanto, a dominância lateral influenciou os modelos de regressão, com canhotos apresentando maior ΔPEC, possivelmente devido à execução preferencial de gestos técnicos unilaterais. Achados semelhantes foram descritos por Paravlic et al., 13 que relataram assimetrias discretas entre membros dominante e não dominante, atribuídas à repetição unilateral de chutes e apoios. Murillo-Ortiz et al. 12 também observaram diferenças sutis em exercícios unilaterais, o que reforça que a dominância e posição em campo geram adaptações biomecânicas funcionais, sem caráter patológico. Assim, o maior ΔPEC observado em canhotos reflete adaptação funcional à dominância técnica, e não deformidade estrutural.

### Assimetrias Laterais

Foram observadas diferenças bilaterais significativas entre PEC, mMPTA e mLDFA, mais evidentes em atletas jovens, o que sugere efeito maturacional na busca por simetria funcional. Esses achados concordam com os de Steffen et al., 9 que relataram redução progressiva das assimetrias angulares com o avanço da idade e o tempo de prática esportiva. A persistência de discrepâncias em zagueiros e canhotos reforça o padrão adaptativo descrito por Paravlic et al. 13 e Murillo-Ortiz et al., 12 nos quais gestos unilaterais e sobrecarga assimétrica promovem ajustes condilares específicos. Clinicamente, tais assimetrias, embora fisiológicas, podem aumentar o risco de sobrecarga articular em esportes de contato com desaceleração e mudanças rápidas de direção.

### Implicações Clínicas

Os achados reforçam a importância do monitoramento bilateral dos ângulos articulares, pois assimetrias podem surgir precocemente e evoluir com a prática esportiva. Steffen et al. 9 e Paravlic et al. 13 destacaram que o acompanhamento sistemático do alinhamento e do equilíbrio entre membros é essencial para prevenir sobrecargas e alterações biomecânicas. Intervenções preventivas devem considerar a posição em campo e a dominância lateral, conforme Murillo-Ortiz et al., 12 visando reduzir a sobrecarga unilateral e promover simetria funcional. Parâmetros como PEC e o ângulo tibiofemoral mecânico ( *mechanical tibiofemoral angle* , mTFA, em inglês) mostraram-se úteis como indicadores sensíveis de sobrecarga articular e detecção precoce de alterações biomecânicas associadas ao risco de lesões.

### Vieses de Medição e Seleção

O estudo apresenta potenciais vieses: a inclusão de atletas de um só clube profissional pode limitar a generalização, e a exclusão de atletas lesionados ou em reabilitação reduz a representatividade clínica. Quanto ao viés de medição, embora o protocolo radiográfico seja padronizado, há possibilidade de variações inter e intraobservador na mensuração dos ângulos, influenciadas por posicionamento e interpretação, apesar do uso de ferramentas digitais e métodos estatísticos para reduzir erros. Tais limitações reforçam a necessidade de interpretação cautelosa e sugerem a realização de estudos multicêntricos, longitudinais e com técnicas automatizadas de mensuração para aprimorar a validade dos resultados.

### Limitações e Perspectivas

Apesar da representatividade da amostra e da robustez estatística, o delineamento transversal limita inferências causais e impede a avaliação da evolução longitudinal dos ângulos articulares. Variáveis adicionais, como o tempo de prática esportiva, o histórico de lesões e as preferências motoras, poderiam enriquecer os modelos. Recomenda-se que estudos multicêntricos e longitudinais associem dados morfológicos a desfechos clínicos como dor, lesão e desempenho.

### Validade Externa e Generalização dos Resultados

A validade externa é restrita pela inclusão de atletas de um só clube profissional de Belém, o que reduz a diversidade cultural e estrutural. Embora a homogeneidade favoreça o controle de variáveis, limita a generalização para atletas de base, amadores, de outras regiões ou do sexo feminino. Assim, os achados devem ser interpretados com cautela fora desse contexto, mas fornecem base relevante para hipóteses futuras e reforçam a necessidade de investigações mais amplas, que aumentem a aplicabilidade clínica das evidências.

## Conclusão

O alinhamento em valgo foi a alteração mais prevalente (68,62%), que predominou entre laterais e meias, com valgo bilateral em (47,05%), o que sugere maior simetria anatômica. O varo ocorreu em (17,64%), principalmente unilateral (E: 5,88%; D: 10,78%), o que indica adaptação funcional assimétrica. O eixo mecânico apresentou correlação negativa com a idade: atletas de até 22 anos exibiram maior prevalência de valgo (72%), associada a PEC >7° e mLDFA < 88°. Atletas com 23 anos ou mais mostraram distribuição mais equilibrada entre valgo (52%) e neutro (43%), o que sugere estabilização progressiva do alinhamento com o envelhecimento. Zagueiros e canhotos apresentaram maior assimetria condilar (ΔPEC), e 68% dos atletas mostraram associação entre o membro dominante e valores menores de PEC (< 7°) ou mMPTA (> 3°), ao passo que o membro de apoio exibiu maior valgo (PEC > 7° e mMPTA > 3°), o que reflete demandas posicionais e biomecânicas específicas. O PEC correlacionou-se negativamente com o mLDFA (D: -68,8%; E: -69,4%) e positivamente com o mMPTA (+60,6% e +60,3% nos lados direito e esquerdo, respectivamente), ao passo que o mTFA apresentou correlação negativa intensa (-99,7%), o que evidencia influência direta na posição do eixo mecânico.
